# Nurses’ Organization of Work and Its Relation to Workload in Medical Surgical Units: A Cross-Sectional Observational Multi-Center Study

**DOI:** 10.3390/healthcare11020156

**Published:** 2023-01-04

**Authors:** Federica Maria Pia Ferramosca, Maddalena De Maria, Dhurata Ivziku, Barbara Raffaele, Marzia Lommi, Maria Ymelda Tolentino Diaz, Graziella Montini, Barbara Porcelli, Anna De Benedictis, Daniela Tartaglini, Raffaella Gualandi

**Affiliations:** 1 Department of Biomedicine and Prevention, University of Rome Tor Vergata, 00133 Rome, Italy; 2 Degree Course in Nursing, UniCamillus International Medical University, 00131 Rome, Italy; 3 Department of Healthcare Professions, Campus Bio-Medico of Rome University Hospital, 00128 Rome, Italy; 4 Degree Course in Nursing, University of Rome Tor Vergata, 00133 Rome, Italy; 5 Local Health Authority Roma 2, 00159 Rome, Italy; 6 Degree Course in Nursing, La Sapienza University, 00185 Rome, Italy; 7 Degree Course in Nursing, University Campus Bio-Medico, 00128 Rome, Italy; 8 Italian Scientific Society for the Direction and Management of Nursing (SIDMI), 10058 Torino, Italy

**Keywords:** hospitals, nurses, workplace, working conditions, workflow, workload

## Abstract

Introduction: Work contexts can affect nurses’ work and work outcomes. Work context factors of nurses, patients, or workflow can modulate nurses’ organization of work and determine increased workloads. Aim: The aim of this research was to analyze relationships between factors regarding the patient, the nurse, workflow, and nurses’ work organization, to investigate whether work organization is related to physical, mental, and emotional workloads, and to explore whether one dimension of workload influences the other dimensions. Methods: We used a cross-sectional design based on the Job Demand-Resources theory. We asked registered nurses, working in nine medical-surgical wards across three hospitals in Italy, to self-report on work organization and workloads regarding randomized shifts over three consecutive weeks. Four scales from the QEEW 2.0 questionnaire were used on an online survey for data collection. multivariable linear regressions with structural equation modelling were tested. The study was approved by the three local Ethics Committees. Results: We received 334 questionnaires regarding 125 shifts worked. Patient complexity (β = 0.347), patient specialties (β = 0.127), adequacy of staffing (β = −0.204), collaboration with colleagues (β = −0.155), unscheduled activities (β = 0.213), supply search (β = 0.141), and documentation (β = 0.221) significantly influenced nurses’ work organization. Nurses’ work organization was significantly related to physical, mental, and emotional nursing workloads. Conclusions: the patient, the nurse, and workflow aspects influence nurses’ work organization and workloads. Healthcare organizations, managers, and nurses should explore work settings to identify work turbulences early and implement strategies to improve nursing work conditions and workloads.

## 1. Introduction

The political, economic, social, and demographic changes in recent years have profoundly influenced the management and development of healthcare systems. These challenges, accompanied by reduced investment in the healthcare workforce and absence of strategic planning, have induced the worldwide “health workforce crisis” [1]. The World Health Organization (WHO) defines the chronic shortage of healthcare workers as one of the most critical obstacles to achieving health and providing effective health services [2]. Therefore, to face the present crisis, it is necessary to invest in well-trained and educated nurses [2], to empower the potential of the present nursing workforce, and improve working conditions to guarantee productivity and responsiveness [3], increase the quality of care, and improve patient experience [4] in a perspective of the ethics of the job well done [5].

Nursing work environments change constantly, grow in complexity, and are defined by unique characteristics among countries and healthcare systems [6]. There is an extensive amount of literature exploring nursing work environments and documenting its influence on patient and nurse outcomes, such as patients’ satisfaction, safety or quality of care, and nurses’ stress, emotional exhaustion, performance, engagement, or absenteeism [7]. These studies gathered nurses’ perceptions regarding their work environments and general effects and have the limit of not focusing on observed tangible situations. Different instruments are available to measure nursing work environments, but they present important limits: most of them evaluate predominantly structure and outcome aspects rather than work processes in specific work contexts, and their generalized validity is questioned due to constant changes in settings [6]. With the intention to expand present knowledge regarding nursing workload and its relationship to work processes and work environments, this research team is contributing to the exploration of the phenomenon with studies identifying antecedents of general workload [8] and specific for the physical, mental, and emotional dimensions of workload [9]. Present findings inform that, in work contexts, aspects related to nursing staffing and skill-mix, patient complexity, number of specialties, or nurse-to-patient ratio, and workflow aspects regarding unscheduled activities, healthcare documentation, or patient isolation may influence perceptions of nurses regarding shift workloads [8,9]. Another variable that might influence nursing workload is nurse’s work organization. In the nursing literature, only a few studies specifically looked at relationships between nurses’ work organization and workload. The importance of the phenomenon, the constant evolution, and variability motivate the need for further studies, especially focusing on the exploration of nursing work processes in inpatient care settings and its short-term outcomes on nurses. For example, little is known on how specific work context aspects facilitate or constrain nurses’ work process or work organization within shifts and whether nurses’ organization of work influences explicit workload dimensions on nurses. This enquiry inspired our research. Through the current study, we want to take a step forward to understand if work context variables influence the organization of nurse’s work and if a consequent disorganization can accentuate the perceived workloads.

In acute care settings, increased care demands, scant human resources, and poor working environments lead to turnover intentions that broaden furtherly the shortage of nurses [10]. When it is impossible to modify care demands and human resources in workplaces, looking at work contexts and correcting disturbances in nurses’ work organization can be a feasible solution to buffer the negative physical or psychological work experiences and workloads. Therefore, healthcare organizations should explore work settings to find new ways to support staff [10].

Exploring nurses’ work organization within work contexts can provide important information to managers to improve work conditions and optimize nursing care. Work organization can be defined as “the way work is structured, distributed, elaborated, and supervised” [11]. The nurses’ work organization has varying interpretations in the literature. It was analyzed in terms of work schedule, models of nursing care, nursing tasks [12], or workflow [13]. Furthermore, it was examined in connection with work and structural characteristics [14], work environment or context [15], organizational aspects [16], or social support at work [17]. Different theoretical frameworks have been adopted in the research on nursing work organization, such as the Donabedian’s framework distributing variables in structure-process-outcome, Galbraith’s theory of contingency approach classifying variables in structure and environment [16], or the Job Demand-Resources theory [18] categorizing working conditions as job demands and job resources. Whatever the theoretical background, the concept of nurses’ work organization was related to aspects of nurses and nursing workforce, patient variability and needs, work contexts, resources, and relationships in work settings.

In this study, nurses’ work organization was interpreted as aspects related to work contexts that interfere with nurses’ work processes. We focused our attention on exploring some work context factors related to nurses (for example the adequacy of staffing or collaboration with colleagues), patients (for example their complexity of care or number of specialties), and workflow (for example unscheduled activities, supply search, healthcare documentation) that can influence the nurses’ work organization. When such context variables are unsatisfactory, they can highlight an inefficient work process characterized by continuous interruptions, unnecessary rework, delays, and unfinished activities [13]. On the contrary, if within a work context the activities are accomplished through an organized workflow, the organization of work may appear appropriate; nurses will be able to respond adequately to changes in the physical, mental, and emotional workload and changes related to patients’ clinical conditions [15]. Therefore, considering that the goal of healthcare systems is to provide efficient and effective care, it is necessary to conduct studies that examine the nurses’ work organization, recognize imbalances early, and allow managers to implement strategies that correct this disorganization to improve nursing workload, satisfaction, and performance, thus guaranteeing a higher quality of patient care and nurse well-being.

**Figure 1:**
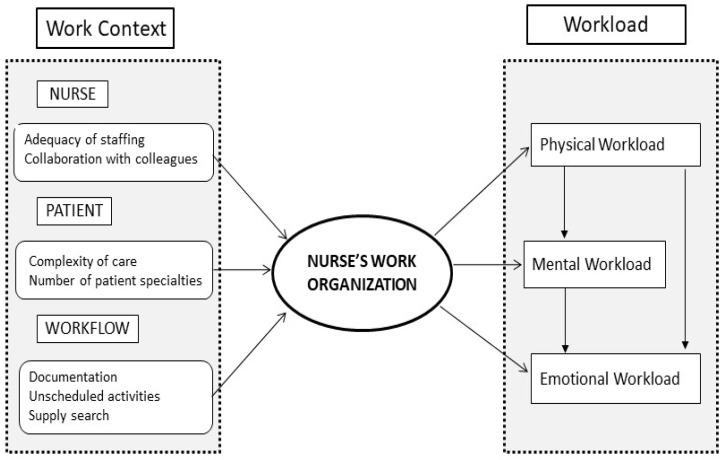
Conceptual model of the study.

The purpose of this study was: (i) to analyze relationships between factors regarding the patient, nurse, workflow, and nurses’ work organization; (ii) to investigate whether work organization is related to physical, mental, and emotional workloads; and (iii) to explore whether one dimension of workload influences the other dimensions. The conceptual model of the study is presented in Figure 1. 

Specifically, we tested the following hypotheses:

**Hypothesis 1.**  *A relationship exists between variables connected to the nurse (independent variables: adequacy of staffing, collaboration with colleagues), the patient (independent variables: complexity of care, number of specialties), and the workflow (independent variable: unscheduled activities, supply search, healthcare documentation) and the nurses’ work organization (dependent variable).*

**Hypothesis 2.**  *A relationship exists between nurses’ work organization (independent variable) and nurses’ perception of physical workload (dependent variable).*

**Hypothesis 3.**  *A relationship exists between nurses’ work organization (independent variable) and nurses’ perception of mental workload (dependent variable).*

**Hypothesis 4.**  *A relationship exists between nurses’ work organization (independent variable) and nurses’ perception of emotional workload (dependent variable).*

**Hypothesis 5.**  *Physical, mental, and emotional workloads are interrelated.*

## 2. Materials and Methods

### 2.1. Design

To conduct this investigation, we used a cross-sectional design. The research followed the STROBE Guidelines for observational studies [19].

### 2.2. Instruments

A survey was prepared to collect nursing perceptions regarding context aspects that influence nurses’ work organization, workloads, and demographic aspects. Nurses were asked to provide some socio-demographic information about gender, age, working experience, and ward they worked in, measured with questions purposefully developed for the study.

Additionally, to measure work context aspects that influence nursing work organization, nurses were asked to provide information about their perception of adequacy of staffing and collaboration with colleagues in the shifts observed. These aspects were measured through single items with a 4-point Likert scale from 0 (not appropriate) to 4 (completely appropriate). Nurses also reported the number of patients cared for in the shift, their different medical specialties, and their complexity of care measured with single items with a 4-point Likert scale (from 0-no complexity to 4-high complexity). Workflow variables such as engagement in unscheduled activities, supply search, and healthcare documentation were captured through single items with a 4-point Likert scale from 0 (no engagement) to 4 (high activity engagement). The use of single-item measurements is recommended in the literature when a concrete construct is evaluated [20].

To measure nurses’ work organization and workloads, we used scales from the Questionnaire on the Experience and Evaluation of Work (QEEW 2.0)© SKB questionnaire [21]. The Work Organization scale consists of six statement items rated on a 4-point Likert scale from 0 (never) to 4 (always), where lower scores refer to better results. The scale is unidimensional and has a good internal consistency (Rho 0.76) [21]. To measure physical workload, we used the Pace and Amount of Work scale. This scale has six items rated on a 4-point Likert scale from 0 (never) to 4 (always), where lower scores refer to lower physical workload. The scale is unidimensional and has a good internal consistency (Rho 0.86) [21]. To measure mental workload, we used the Mental Workload scale. This scale has four items rated on a 4-point Likert scale from 0 (never) to 4 (always), where lower scores refer to lower mental workload. The scale is unidimensional and has a good internal consistency (Rho 0.81) [21]. To measure emotional workload, we used the Emotional Workload scale. This scale has five items rated on a 4-point Likert scale from 0 (never) to 4 (always), where lower scores refer to lower emotional workload. The scale is unidimensional and has a good internal consistency (Rho 0.80) [21]. These scales were already tested within the Italian population.

### 2.3. Population and Sampling

This study involved registered nurses working in nine medical-surgical wards of three teaching hospitals in Italy. The choice of hospitals was of convenience; the authors of this research were employed in these hospitals as research nurses and were involved in data collection. The purposive sampling strategy was used for the selections of wards; to be included the units must offer nursing care in assisting major surgery and general medicine patients, excluding units that cared for COVID-19 infected patients. All nurses working in the included wards were invited to participate in the study, based in inclusion and exclusion criteria. A total of 185 nurses were eligible to participate in the study, among these, only 30 nurses denied consent for participation.

### 2.4. Inclusion and Exclusion Criteria

Only full-time registered nurses directly involved in patient care and working in the ward for at least 2 months were included in the research. We excluded float nurses and nurses working extra time or in additional shifts.

### 2.5. Data Collection

At the beginning, the researchers approached the three hospital nurse executives and the nurse managers of the selected wards to explain the aims of the research and for acquiring their approval of the study. Then, different meetings were organized to reach all nurses working in the wards, to explain the study and the required involvement. Specifically, nurses were invited to self-complete a questionnaire about work organization and workloads at the end of a working shift, randomly chosen from mornings and afternoons over three consecutive weeks during 2021. Nurses received a Google Forms link in their institutional email and were free to answer the entire questionnaire or parts of it.

### 2.6. Sample Size and Power

A sample size of 311 surveys could achieve 95% power to conduct a multivariable linear regression analysis using 10 predictors with an anticipated effect size of 0.08 and a level of significance of *p* < 0.001. Nevertheless, 334 surveys were collected for a more stable analysis. The estimation of sample size was performed with G*Power 3.1. 

### 2.7. Data Analysis

Sample characteristics and variables studied were described by means, standard deviations (SD), frequencies, or percentages. Preliminary analyses were conducted to check for missing values, outliers, and to test for multicollinearity and normality assumptions [22].

To test our hypothesis, we fitted multivariable linear regression models using full structural equation modelling (SEM) and implementing maximum likelihood (ML) estimation [23]. The choice of the independent variables was theoretically driven. Variables related to the nurse (perception of adequacy of staffing, collaboration with colleagues), the patient (complexity and number of specialties), and the workflow (unscheduled activities, documentation, supply search), were specified as independent variables in the model, whereas nurses’ work organization was specified as the dependent variable. Two separate SEMs were tested. The first model aimed to identify the relationship among (i) variables related to the nurse, the patient, and the workflow, and the nurses’ work organization and, (ii) nurses’ work organization and nurses physical, mental, and emotional workloads. The second model aimed to identify the relationships among (iii) the three different dimensions of workload: physical on mental and emotional workloads, and metal on emotional workload. Since the independent variables are correlated, they were included together in the SEMs to control the reciprocal influence [18]. To evaluate model fit, several goodness-of-fit indices [23] were used. Regression parameters are shown as unstandardized and standardized coefficients. The coefficient of determination (R^2^) was also reported. Statistical tests were two-sided; *p*-values < 0.05 were considered significant. Preliminary and descriptive analyses were conducted in SPSS v. 26.0, the SEMs were estimated in MPLUS v. 8.4.

### 2.8. Ethical Considerations

The study was approved by the three local Ethics Committees. The researchers approached nurses individually and collected the written informed consent. Those who refused to sign the informed consent were excluded from the study. Anonymity was guaranteed by the attribution of a unique numeric code to each participant [24]. Data access was restricted to the research team. 

## 3. Results

We received 334 completed surveys from nurses working in 125 randomized shifts (185 surveys in morning and 149 in afternoon shifts), with a response rate of 91.7%. No missing data, outliers, or multicollinearity on regression analyses were recorded. Normal distribution and linearity were satisfactory.

### 3.1. Sample Characteristics

The descriptive analysis of variables studied are presented in Table 1. The mean age of nurses that answered the survey was 35 years; 38% had more than 10 years of working experience. The majority of nurses reported taking care of patients with moderate and high complexity needs (mean 2.9, SD 0.8), and were more frequently from two or more specialties (mean 2.5, SD 1.3). They reported mostly a good collaboration with colleagues (mean 2.9, SD 0.8) and 36% reported to work on understaffed shifts. Nurses reported a mean score of 1.5 (SD 1.1) in supply search load, a mean of 1.7 (SD 1.2) on unscheduled activities, and 58.7% reported a high load on healthcare documentation. The mean scores on work organization were 47.0 (SD 17.6) and means for physical workload were 45.7 (SD 23.7), indicating an average load level, whereas higher scores were reported on emotional workload (mean 55.2, SD 16.3). The highest workload reported was mental, with a mean score of 88.4 (SD 17.0).

### 3.2. Variables Related to the Nurse’s Work Organization

To test hypothesis one, two, three, and four, we accomplished one model. In this model, we found that patient complexity (*p* < 0.001), patient specialties (*p* = 0.006), adequacy of staffing in the shift (*p* < 0.001), collaboration with colleagues (*p* = 0.001), unscheduled activities (*p* < 0.001), healthcare documentation (*p* < 0.001), and supply search (*p* = 0.004) were significantly related to nurse’s work organization. Specifically, increasing the complexity of patients, the number of patient specialties, the amount of healthcare documentation, the unscheduled activities, and continuous search for supplies disrupts nurse’s organization of work. Additionally, working in shifts that are understaffed and with poor collaboration will lead to disruption of nurse’s work.

The analysis regarding the hypothesis two, three, and four documented relationships between nurse’s work organization and all the three dimensions of nurse’s workload. When nurses perceive disruptions on their work organization, they report increased physical workloads (*p* < 0.001), amplified emotional workloads (*p* < 0.001), and higher mental loads (*p* < 0.001). For further details, see Table 2. The goodness of fit statistics for this model were as follows: χ2 (314, N = 334) = 566.468, *p* < 0.001, CFI = 0.928, TLI = 0.918, RMSEA = 0.0489 (90% CI = 0.043–0.055), *p* = 0.586, SRMR = 0.064.

### 3.3. Relationships between Different Dimensions of Nurse’s Workloads

To assess reciprocal effects among the three dimensions of nursing workload and validate the fifth hypothesis we tested a separate model. We found that physical workload was significantly related to mental (*p* < 0.001) and emotional workloads (*p* < 0.001), and no relationships were identified between mental and emotional workloads (*p* = 0.320). When nurses report an increment on physical load, they will report as well higher mental and emotional loads. For further details, see Table 3. The goodness of fit statistics for this model were as follows: χ2 (82, N = 334) = 191.353, *p* < 0.001, CFI = 0.955, TLI = 0.942, RMSEA = 0.050 (90% CI = 0.052–0.075), *p* = 0.032, SRMR = 0.066.3.1.

## 4. Discussion

The aim of this study was (i) to analyze relationships between factors regarding the patient, nurse, workflow, and nurses’ work organization, (ii) to investigate whether work organization is related to physical, mental, and emotional workloads, and (iii) to explore whether one dimension of workload influences the other dimensions. We found different significant effects that contribute importantly to the literature on nursing work organization and workload.

The findings of our study come mostly from responses of female nurses, the majority with less than 2 years or more than 10 years of work experience. Work experience influences the definition and assignment of tasks, the planning, and the methods used to carry them out [25]. Less experienced nurses might have fewer skills and autonomy in meeting the patients’ needs, might engage in additional activities, or consult with colleagues, and might perceive an increased volume of work and may be more susceptible to higher workloads [8]. In our sample, the great heterogeneity of work experience among nurses did not allow us to significantly confirm a relationship between work experience and nurse’s work organization. Therefore, more studies are needed to explore such relationships. Additionally, despite the medium scoring levels of nurse’s work organization, we were able to identify different significant relationships between work context variables and nurse’s work organization, as described below.

Concerning the first hypothesis, there are only a few studies in the literature that explore relationships between work context factors and nurses’ work organization based on work processes. Regarding the variables related to patients, the complexity of care and the number of patient specialties have a significant effect on the nurses’ work organization, where complexity of care presents a greater impact. We deduced that patients’ complexity interferes with the nurses’ work organization because the nurse must continuously modify the plan of care based on decisions about continuous patient health changes. To support our reflection, a previous review on organization of work [12] connects the complexity of care with the amount of nursing care needed and staffing resources, aspects that influence the work organization. In addition, a greater number of patient specialties can lead to continuous work interruptions, increased requests for information, and interface with different medical teams that could disrupt the work process [26], which could generate changes in the organization of work. To our knowledge, there are no studies that specifically evaluated the effects of the number of patient specialties and patients’ complexity on nurses’ work organization; therefore, our findings increase knowledge on this aspect of nursing work. Previous studies have documented that these variables significantly influenced nursing workload [8] and nursing care activities [27]. Knowing that patient specialties and patients’ complexity disrupt nurses’ work organization is important to managers because it can guide them in defining staffing policies regarding appropriate number of staff in the shift, and it is important to nurses because it can help them to better organize division of work and allocation of care activities.

Adequate staffing and collaborative relationships are essential for work contexts. Human resources are important for responding to complex patient needs [2,3,28], to ensure the provision of high-quality nursing care [2,3,29], and to reduce hospitalization and mortality [30]. Our study highlighted that adequacy of staffing on the shift influences nurses’ work organization. We hypothesized that working in understaffed shifts will increase the number of activities that nurses should perform, probably facing additional interruptions, which can have a negative effect on their organization of work. In addition to adequacy of staffing, it is also important to pay attention to the collaboration of nurses. Nurse collaboration includes sharing, teamwork, and respect [31]. In the previous literature, good collaboration with colleagues within shifts has shown to improve the effectiveness of services and the quality of care provided [32]. A work environment characterized by difficult interactions and contrasts between nurses reduces collaboration in the work team and can be translated in reduced job performance, productivity, and nurses’ psychological health [33]. We deduce that when nurses have poor collaboration with each other, there are difficulties in organizing and sharing activities and continuous delays in tasks execution, and this may determine an alteration of the organization of work. Therefore, to improve nurses’ work organization in care settings, it is necessary to strengthen employers’ engagement in team building and promote effective collaboration in work contexts.

Focusing attention on variables related to workflow, the results of our study describe that unscheduled activities, volume of healthcare documentation, and supply search can affect the nurses’ organization of work. In a recent review [34], associations between uncertain activities, disjointed supply locations, and disruption of temporal and spatial workflows were recognized. Documentation in healthcare settings amplifies the perception of time pressure [35] and the use of technology in electronic health records was described as an element that interferes with the organization of work [12]. Disorganization of nurses’ work was frequently described as a reason for reduced quality of care [10], decreased efficiency at work, and cognitive load [34]. Therefore, these turbulences in workflow should be addressed with strategies on reorganization of work environments to reduce interruptions and improve nursing care quality.

Concerning the second, third, and fourth hypotheses, nurses’ work organization was proven to influence all three workload dimensions. Recently, nursing management research has focused on the description of work context factors that influence nursing workload in general [8], or have associations with physical, emotional, or mental nursing workloads [9,36]. In the present study, the nurses’ organization of work affects all the three dimensions of workload, influencing more the physical and emotional loads. We hypothesize that the physical load is greater in disorganized work contexts because nurses make greater efforts to perform additional tasks and with a faster pace. A similar work context will ask nurses to be more focused and more intensely involved in decision-making, generating increased mental loads. Additionally, a turbulent organization of work will frustrate nurses since they will not be able to fully respond to all patient needs, and this will increase the perception of emotional load. In the nursing literature, a few studies specifically described the relationship between nurses’ work organization and workload, from the work process viewpoint. In one study [14], work complexity and task uncertainty were seen as characteristics of nursing work organization and were predictive of nursing workload. Chaotic environments might increase physical and cognitive workloads [34]. Previous research documented that patient complexity, adequacy of staffing, unscheduled activities, healthcare documentation, and patients’ specialties directly influenced perceived nursing workload [8]. In the present study, these variables were also found to disrupt nurses’ work organization. This information is essential for healthcare managers to actuate strategies focusing on variables that are contemporarily related with work organization and nursing workload, doubling benefits, and improving work outcomes. Therefore, it is fundamental to continue to study aspects that interfere with nurses’ work organization to ensure better work environments and reduce workload.

Our findings regarding the fifth hypothesis provide insight into the influence of one nursing workload on another, delving into an aspect little explored in the previous literature. We found that physical workload affects emotional and mental workloads and that there is no relationship between mental and emotional workloads. In the literature, we found only two studies that discussed connections between different nursing workloads. Increased physical exertion requires mental demands that can reduce nurses’ concentration [37]. Covariation also exists between physical and emotional workloads. Working on understaffed shifts will increase physical workload on nurses, which in turn can produce emotional strain [38]. Furthermore, we found no relationships between mental and emotional workloads. We hypothesize that the reason is nested in the different domains involved: the mental load requires more decision-making and cognitive concentration, whereas the emotional load is more related to feelings and emotions regarding relationships with patients or colleagues in the work contexts. Therefore, the new knowledge introduced by our findings that physical workload generates greater mental and emotional loads is essential to nurse management research. When top and middle managers in healthcare organizations are setting policies to improve nurses’ workload, we suggest they carefully choose interventions that will determine larger effects, knowing that reducing physical strain will improve mental and emotional loads.

Our study can be considered the first study that tries to explain determinants of nurses’ work organization and its effects on physical, mental, and emotional workloads. Understanding what causes nurses’ work disruption and disorganization is essential for nurses and managers. If nurses and managers know what variables related to the patient, nurses, and workflow generate turbulence at work, they will be able to, based on specific role competences, adjust work environments and work collaboration to reduce such aspects. Nurses who perceive work environments as organized and positive will be more productive through better performance and will perceive organizational well-being [39].

### 4.1. Strengths and Limitations

This study contributes significantly to research in the field of nursing work organization and workload. To our knowledge, the study is the first to explore antecedents of nurses’ work organization focused on work processes and how it affects nursing workload in all the three dimensions. Additionally, this study amplifies knowledge on nursing workload and provides evidence that one dimension of workload can influence others. Considering that perceptions can change depending on the shift and work context, to generalize our findings, this study gathered perceptions of nurses in specific shifts worked, in medical-surgical departments, both in public and private hospitals.

However, the present study has also some limitations. The only way to acknowledge how nurses perceive their work is through self-assessment questionnaires. This could generate a response bias toward social desirability. Additionally, the cross-sectional design of the study hinders the identification of cause-effect relationships. We tried to bypass this limitation by asking nurses to report their perceptions of work organization and workload regarding specific shifts worked repeatedly in three consecutive weeks. Besides design, the analysis was performed at an individual level and the identification of possible unit and hospital aggregated influencing aspects were not analyzed. A larger sample will allow the exploration of these aspects at multiple levels, including units and hospitals and allowing the identification of other influencing aspects not considered in the present study.

### 4.2. Recommendations for Further Research

Future research is needed to confirm our findings and to explore other workflow variables that can influence nurses’ work organization. Moreover, researchers could consider exploring the efficacy of interventions on reorganization of work contexts, resource allocation, individual nurses’ work arrangements, and organization of teamwork on the shift. Additionally, researchers should measure efficacy of these interventions on different dimensions of nursing workloads.

### 4.3. Implications for Nursing Practice

The disruption of work organization and work processes can determine work pressure and stress on nurses, and interfere with work performance [17] and the quality of patient care [10]. Organizations need to find new ways to support weary nursing staff and promote their well-being at work [2,3,10]. Building healthy work contexts is essential for nursing workforce retention.

Chief executives and managers could use our results to set policies regarding improvements on work contexts and workflows by adopting some proposed interventions:

- Redesign work systems to support nurses by adjusting physical work environments (better distribution of spaces and material resources) by guaranteeing appropriate human resources (adequacy of staffing, proper work schedule, and balance of skill-mix), and by encouraging cooperation and collaboration among multidisciplinary teams through professionalism [40].
- Stimulate nurses’ participation as main actors in the identification of appropriate solutions to improve nurses’ work organization, to sustain the change process through active decision-making and peer support, and foster well-being [34].
- Codesign, in collaboration with multidisciplinary teams, interventions to eliminate redundant documentation [13] and better program activities to manage job demands and resources, improve workflow, remove additional stress on nurses, and allow them to dedicate more time to patient care.

## 5. Conclusions

This study showed that work context factors related to patients, nurses, and workflow can influence nurses’ work organization and that work organization has an impact on physical, mental, and emotional dimensions of nursing workload. Additionally, we discovered that one dimension of workload can influence others. Research on the organization of nursing processes at work and its effects on workload dimensions is scarce; therefore, future studies on this topic are recommended. Healthcare managers should consider our results and address them on policy regarding staffing to improve nurses’ organization of work, mitigate its effect on the nursing workload and enhance well-being.

## Figures and Tables

**Table 1:** Descriptive characteristics of the sample and variables studied (N = 344).

Variable	N (%)	Mean ± SD (Range)
**Gender**		
Female	282 (84.4)	
Male	52 (15.6)	
**Age**		34.89 ± 9.14 (22–64)
**Hospitals**		
1	205 (61.3)	
2	69 (20.7)	
3	60 (18.0)	
**Units**		
Surgery	5 (55.5)	
Medicine	4 (44.5)	
**Number of Surveys by Shifts**		
Morning	185 (55.4)	
Afternoon	149 (44.6)	
**Work Experience in Months**		
0–24	101(30.2)	
25–60	50 (15.0)	
61–120	56 (16.8)	
>121	127 (38.0)	
**Number of Patient Specialties**		2.5 ± 1.3 (1–7)
≤2	182 (54.5)	
3–4	121 (36.2)	
≥5	31 (9.3)	
**Patient Complexity**		2.9 ± 0.8 (0–4)
Not at all/A little	12 (3.6)	
On Average	107 (32.0)	
Enough/A lot	215 (64.4)	
**Adequacy of Staffing in the Shift**		1.8 ±1.0 (0–4)
Not at all/A little	121 (36.2)	
On Average	141 (42.2)	
Enough/A lot	72 (21.6)	
**Collaboration with Colleagues**		2.9 ± 0.8 (0–4)
Not at all/A little	13 (3.9)	
On Average	98 (29.3)	
Enough/A lot	223 (66.8)	
**Health Care Documentation Load**		2.6 ± 1.0 (0–4)
Not at all/A little	37 (11.1)	
On Average	101 (30.2)	
Enough/A lot	196 (58.7)	
**Supply Search Load**		1.5 ± 1.1 (0–4)
Not at all/A little	147 (44.0)	
On Average	115 (34.4)	
Enough/A lot	72 (21.6)	
**Unscheduled Activities**		1.7 ± 1.2 (0–4)
Not at all/A little	160 (47.9)	
On Average	89 (26.6)	
Enough/A lot	85 (25.5)	
**Work Organization**		47.0 ± 17.6 (0–100)
**Physical Workload**		45.7 ± 23.7 (0–100)
**Mental Workload**		88.4 ± 17.0 (33.3–100)
**Emotional Workload**		55.2 ± 16.3 (20–100)

*Notes*: Hospital 1, private, is situated in Central Italy, data were collected in February 2021; Hospital 2, public, is situated in the South of Italy, data were collected in September 2021; Hospital 3, public, is situated in Central Italy, data were collected in September 2021.

**Table 2:** Multivariable regression effects of the patient, nurse, and workflow variables on nurses’ work organization and effects of nurses’ work organization on perceived physical, mental, and emotional workloads (N = 334).

Model 1: Work Organization	b	β	SE	*p*-Value	95% CI
*Patient variables*					
Patient complexity	0.145	0.347	0.050	<0.001	0.248 0.446
Patient specialties	0.035	0.127	0.047	0.006	0.035 0.218
*Nurse variables*					
Adequacy of staffing	−0.070	−0.204	0.049	<0.001	−0.301 −0.108
Collaboration with colleagues	−0.066	−0.155	0.049	0.001	−0.248 −0.061
*Workflow variables*					
Unscheduled activities	0.062	0.213	0.052	<0.001	0.111 0.314
Healthcare documentation	0.076	0.221	0.049	<0.001	0.125 0.317
Supply search	0.048	0.141	0.049	0.004	0.045 0.236
**Relationships between nurses’ work organization and perceived workloads**					
Work organization on physical workload	1.537	0.847	0.029	<0.001	0.789 0.904
Work organization on mental workload	0.361	0.276	0.060	<0.001	0.159 0.431
Work organization on emotional workload	0.522	0.401	0.061	<0.001	0.283 0.557

*Abbreviations*: b, unstandardized coefficient; β, standardized coefficient; SE, standard error; CI, Confidence Interval. *Notes*: R^2^, coefficient of determination; work organization R^2^ = 0.572, *p* < 0.001; work organization on physical workload R^2^ = 0,717, *p* < 0.001; work organization on emotional workload R^2^ = 0.161, *p* = 0.001; work organization on mental workload R^2^ = 0.076, *p* = 0.021.

**Table 3:** Effects of one dimension of nurses’ workload on the others (physical, mental, and emotional workloads) (N = 334).

Model 2	b	β	SE	*p-*Value	95% CI
Physical workload on mental workload	0.232	0.164	0.059	<0.001	0.048 0.279
Physical workload on emotional workload	0.515	0.351	0.059	<0.001	0.235 0.468
Mental workload on emotional workload	0.070	0.068	0.068	0.320	−0.066 0.201

*Abbreviations*: b, unstandardized coefficient; β, standardized coefficient; SE, standard error; CI, Confidence Interval. *Notes*: R^2^, coefficient of determination; Physical workload on other workloads R^2^ = 0.158, *p* = 0.001.

## Data Availability

The data presented in this study are available on request from the corresponding author. The data are not publicly available due to privacy restrictions.
